# Trends in dietary intake and food sources of long-chain polyunsaturated fatty acids among Korean adults between 2007 and 2018

**DOI:** 10.4178/epih.e2023069

**Published:** 2023-08-01

**Authors:** Jae Eun Shim, Youngmi Lee, SuJin Song

**Affiliations:** 1 Department of Food and Nutrition, Daejeon University, Daejeon, Korea; 2 Department of Food and Nutrition, Myongji University, Yongin, Korea; 3 Department of Food and Nutrition, Hannam University, Daejeon, Korea

**Keywords:** Fatty acids, Alpha-linolenic acid, Eicosapentaenoic acid, Docosahexaenoic acids, Linoleic acid, Korea

## Abstract

**OBJECTIVES:**

This study examined trends in the dietary intake and food sources of long-chain polyunsaturated fatty acids (LC-PUFAs) in Korean adults from 2007 to 2018.

**METHODS:**

In total, 46,307 adults (aged 19-64 years) were selected from the 2007-2018 Korea National Health and Nutrition Examination Surveys. Dietary data were obtained using 24-hour dietary recall. Intake levels and food sources of LC-PUFAs, including alpha-linolenic acid (ALA), eicosapentaenoic acid (EPA), docosahexaenoic acid (DHA), and linoleic acid (LA), were evaluated across the survey years and assessed based on compliance with dietary recommendations over the 2007-2018 period. Linear trends in LC-PUFAs intake levels over time were examined through multiple linear regression analysis.

**RESULTS:**

From 2007 to 2018, ALA and LA consumption increased from 1.1 g (0.5% of energy) to 1.4 g (0.6% of energy) and from 8.6 g (3.9% of energy) to 10.0 g (4.5% of energy), respectively. EPA intake decreased from 0.14 g to 0.12 g, and EPA+DHA intake showed a decreasing trend. The proportion of individuals who did not meet the recommended intake of EPA+DHA (250 mg/day) increased (64.4 to 68.4%). Regarding changes in food sources of ALA and LA, the contributions from mayonnaise, eggs, and bread increased, while those from plant food sources decreased. Among food sources of EPA and DHA, anchovy, saury, and Atka mackerel showed lower contributions over time.

**CONCLUSIONS:**

Our findings suggest that strategies to encourage the consumption of EPA and DHA from healthy food sources are necessary to improve cardiovascular health in the Korean population.

## GRAPHICAL ABSTRACT

[Fig f2-epih-45-e2023069]

## INTRODUCTION

Non-communicable diseases have emerged as a major global public health concern, with their contribution to mortality and disability rapidly increasing over the past 30 years [1]. In particular, cardiovascular disease (CVD) is the leading cause of death worldwide. The highest proportion of CVD deaths can be attributed to modifiable dietary risk factors and diet-related metabolic risk factors, including high systolic blood pressure and elevated total and low-density lipoprotein (LDL) cholesterol levels [2].

The type of dietary fatty acid consumed primarily influences blood lipid and lipoprotein profiles [3-5]. Saturated fatty acid (SFA) intake has been a particular focus of dietary recommendations aimed at reducing the risk of CVD [6]. However, the health impacts of SFA are contingent on the nutrients that replace it isocalorically [7]. Decreasing SFA intake by substituting it with carbohydrates and unsaturated fatty acids has been demonstrated to lower total cholesterol and LDL cholesterol levels in the blood [7]. While an increase in carbohydrate intake can lead to a rise in triglycerides, it is beneficial to replace SFA with unsaturated fatty acids. The most effective substitution is with polyunsaturated fatty acids (PUFAs).

The consumption of long-chain polyunsaturated fatty acids (LC-PUFAs), such as alpha-linolenic acid (ALA) and linoleic acid (LA), is necessary to supply essential fatty acids, which cannot be synthesized by the human body. Furthermore, meta-analyses of epidemiological studies have indicated that intake of LC-PUFAs, including eicosapentaenoic acid (EPA) and docosahexaenoic acid (DHA), is associated with a reduction in CVD risk [8,9]. Therefore, LC-PUFAs intake is important for both ensuring adequate intake of essential nutrients and replacing SFA intake to lower CVD risk. However, population-based data indicate that a reduction in SFA intake may not result in an increase in PUFAs intake. A systematic review of national dietary surveys and population-based studies on the dietary intake of fatty acids revealed that SFA intake is associated with total fat intake, but not with PUFAs intake [10]. Additionally, prior studies in European countries and the United States have shown that the consumption of omega-3 and omega-6 fatty acids does not reach the recommended levels [11,12].

In a prior study examining trends in fat and fatty acid consumption among Korean adults, it was found that the rise in dietary fat intake over the past decade was primarily due to an increase in SFA consumption. Meanwhile, changes in the intake of PUFAs, especially omega-3 fatty acids, were negligible [13,14]. As CVD remains the leading cause of death in Korea, it is crucial to identify shifts in the intake levels and food sources of LC-PUFAs to propose strategies that promote cardiovascular health in this population. However, detailed quantitative information on the dietary intake and sources of LC-PUFAs in Korea has been scarce. Therefore, this study aimed to investigate the trends in LC-PUFAs consumption among Korean adults from 2007 to 2018 and to analyze the changes in food sources that are associated with these intake trends, categorized by the type of LC-PUFAs.

## MATERIALS AND METHODS

### Data and participants

The Korea National Health and Nutrition Examination Survey (KNHANES) is an annual surveillance initiative carried out by the Korea Disease Control and Prevention Agency. Its purpose is to evaluate the nutritional health status and associated health behaviors among Koreans. The survey participants are recruited from a nationally representative sample of non-institutionalized Korean individuals who are 1 year of age or older. This selection process utilizes multistage stratified cluster sampling. The survey is cross-sectional and comprises a health interview survey, a health examination survey, and a nutrition survey. More detailed information about the survey is available elsewhere [15].

This study utilized data from the fourth (2007-2009), fifth (2010-2012), sixth (2013-2015), and seventh (2016-2018) KNHANES. Among the 49,513 Korean adults aged 19-64 years who participated in the 2007-2018 KNHANES nutrition survey, we excluded those who (1) had a daily energy intake of less than 500 kcal or more than 5,000 kcal, (2) lacked information on household income, or (3) were pregnant or breastfeeding. Consequently, the data analysis included a total of 46,307 adults, comprising 19,715 men and 26,592 women. The KNHANES datasets used in this study are publicly accessible in the KNHANES website repository (https://knhanes.kdca.go.kr/knhanes/sub03/sub03_02_05.do).

### Assessment of long-chain polyunsaturated fatty acids intake and food sources

In this study, the LC-PUFAs examined included ALA, EPA, DHA, and LA. Data on dietary intake were gathered using 1-day 24-hour dietary recall, which was part of the nutrition survey. A fatty acid database of common Korean foods was utilized to assess the intake of LC-PUFAs and identify their food sources [16]. This database, developed in 2014, was designed to evaluate the consumption of dietary fatty acids among Koreans participating in the KNHANES. It contains the fatty acid content of 5,144 food items, derived from both domestic and foreign sources. The intake levels of dietary fatty acids for individuals participating in the sixth and seventh KNAHNES were computed using this database. The methodology for developing this database is elaborated upon elsewhere [16].

The fourth and fifth KNHANES were conducted prior to the establishment of the fatty acid database. To estimate the intake of LC-PUFAs based on dietary data from these surveys, the information on fatty acid content per 100 g of foods appearing in the sixth KNHANES dataset was primarily used [14,17]. Among 3,193 food items (100%) listed in the fourth and fifth KNHANES datasets, 68.8% could be matched to foods included in the sixth dataset (n=2,196). For the food items not present within the sixth KNHANES data (n=997), the fatty acid content was substituted with calculated or imputed fatty acid content values from similar Korean foods (n=922) or from the U.S. Department of Agriculture’s fatty acid database (n=39). Foods with very low fat content, as well as those lacking available content data, were assigned a fatty acid content of zero (n=36). For composite food items, such as sandwiches, pizza, hamburgers, or fried rice with shrimp and vegetables, the fatty acid content corresponding to the weight of the primary ingredient was used.

The fourth, fifth, sixth, and seventh KNHANES data were used to evaluate trends in the consumption of LC-PUFAs across survey years by gender. The intake of LC-PUFAs was measured in grams per day and as a percentage (%) of energy. Additionally, trends in adherence to the recommended levels for each LC-PUFA were assessed across the survey years by gender. These recommended levels were based on the acceptable macronutrient distribution ranges (AMDRs) for fatty acids, as published by the Food and Agriculture Organization of the United Nations (FAO) in 2010 [6]. The AMDRs include > 0.5% of energy for ALA, 0.25-2.00 g/day for EPA+DHA, 2.5-9.0% of energy for LA, and 6-11% of energy for LC-PUFAs. Data from the fourth (2007-2009) and seventh (2016-2018) KNHANES were used to identify changes in primary food sources based on the type of LC-PUFAs. In these survey data, the top 10 food sources of each LC-PUFA were selected, differentiated by gender. The major food sources were identified based on the contributing percentages of food items. These percentages were calculated by dividing the amount of each LC-PUFA within a food item by the total intake of each LC-PUFA from all food items consumed in the study population. The food items were then ranked from highest to lowest based on their contributing percentage.

### Statistical analysis

All statistical analyses were conducted using SAS version 9.4 (SAS Institute Inc., Cary, NC, USA). These analyses considered the multistage complex sampling design effect and utilized appropriate sampling weights. The PROC SURVEY procedures within the SAS software were used to generate estimates for the entire Korean adult population based on the representative survey sample. The LC-PUFAs intake values are presented as means and standard errors (SEs) over the survey years. Adherence to the recommended levels for each LC-PUFA is expressed in the form of percentages across survey years. The major food sources according to the type of LC-PUFAs are expressed as the contributing percentage of each food item. Linear trends in LC-PUFAs intake over time were compared using the multiple linear regression model after adjusting for gender, age, area of residence, household income, and total energy intake, where applicable. Differences in the adherence to the AMDRs for each LC-PUFA over the survey years were tested using the chi-square test. A p-value of less than 0.05 was considered to indicate statistical significance.

### Ethics statement

The KNHANES was officially approved by the Institutional Review Board of the Korea Disease Control and Prevention Agency (No. 2007-02CON-04-P, 2008-04EXP-01-C, 2009-01CON-03-2C, 2010-02CON-21-C, 2011-02CON-06-C, 2012-01EXP-01-2C, 2013-07CON-03-4C, 2013-12 EXP-03-5C, and 2018-01-03-P-A). All participants provided written informed consent.

## RESULTS

### Characteristics of study participants

Table 1 presents the characteristics of the study participants over the course of the survey years. Approximately 52% were men, and an average age of study participants was 41.3 years (SE, 0.1). Over the survey years, significant increases were observed in the proportions of women, participants aged 50-64 years, and those with a higher household income (p<0.001). The level of energy intake varied significantly over the survey years (p<0.001). The energy intake of the study population demonstrated an upward trend from 2007 to 2015, but it has declined since. Increasing trends were observed in the percentage of energy derived from fat and protein over the survey years, while the percentage of energy from carbohydrates significantly decreased over the same period.

### Trends in long-chain polyunsaturated fatty acids intake from 2007 to 2018

Tables 2 and 3 present the trends in LC-PUFAs intake from 2007 to 2018, in grams per day and as a percentage of energy, respectively. During this period, significant increases were noted in the consumption of ALA and LA (p<0.001). These significant trends in ALA and LA intake, both in grams and as a percentage of energy, were observed in both men and women. In contrast, the intake of EPA+DHA showed a downward trend among all participants. Changes in EPA+DHA intake over time were evident in men, but not in women. While EPA intake decreased, DHA intake, when evaluated as a percentage of energy, showed no significant change over time among all participants. LC-PUFAs intake increased significantly from 10.1 g (4.62% of energy) in 2007-2009 to 11.7 g (5.26% of energy) in 2016-2018.

**Figure 1.**
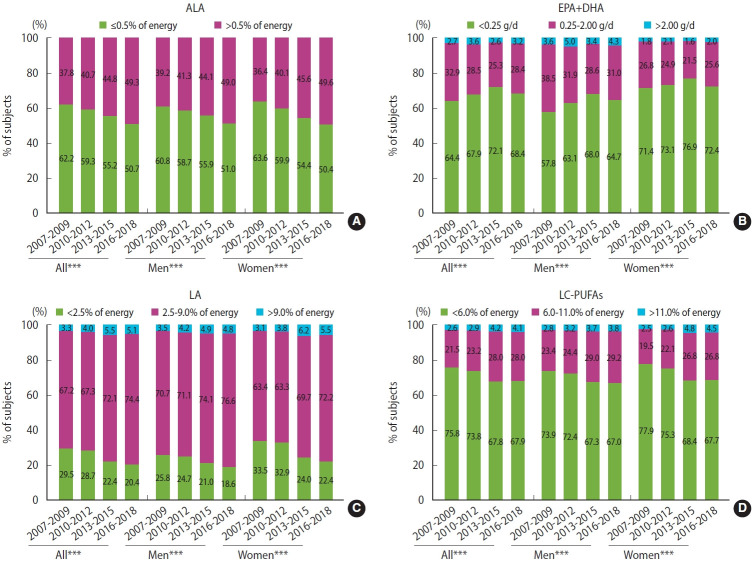
Trends in adherence to dietary recommendations (A: ALA, B: EPA+DHA, C: LA) for the intake of long-chain polyunsaturated fatty acids (LC-PUFAs; D) from 2007 to 2018^1,2,3^. ALA, alpha-linolenic acid; DHA, docosahexaenoic acid; EPA, eicosapentaenoic acid; LA, linoleic acid. ^1^The statistical analysis accounted for the complex sampling design effect and included an appropriate sample weight. ^2^Dietary recommendations include >0.5% of energy for ALA, 0.25-2.00 g/d for EPA+DHA, 2.5-9.0% of energy for LA, and 6-11% of energy for LC-PUFAs. ^3^Differences in the adherence to dietary recommendations for each LC-PUFA across the survey years were tested using the chi-square test. ^***^p<0.001.

Figure 1 illustrates changes in the proportions of study participants based on their intake relative to the AMDRs of LC-PUFAs from 2007 to 2018. An increase was observed in the percentage of participants who consumed ALA and LA above the recommended intake levels throughout the survey years (p<0.001). The proportion of individuals who consumed EPA+DHA below the recommended level (0.25 g/day) rose from 64.4% in 2007-2009 to 68.4% in 2016-2018 (p<0.001). Regarding LC-PUFAs intake, the proportion of participants who met the recommended level (AMDR 6-11% of energy) significantly increased from 2007 to 2018 for both men and women (p<0.001). However, approximately 68% of study participants did not meet the AMDR for LC-PUFAs in 2016-2018.

### Changes in major food sources of long-chain polyunsaturated fatty acids

Table 4 presents the observed changes in the primary food sources of LC-PUFAs from the fourth (2007-2009) to the seventh (2016-2018) KNHANES. The dietary contributions to ALA and LA intake showed increases in mayonnaise, eggs, and bread and decreases in plant-based food sources like tofu, soybeans, and plant oils. For EPA and DHA, food sources such as anchovies, saury, and Atka mackerel demonstrated reduced contributions in the seventh survey data relative to the fourth survey data.

## DISCUSSION

In this study, we observed changes in the intake of LC-PUFAs among Korean adults and compared these changes to population data used in systematic reviews. These reviews were instrumental in establishing fat and fatty acid requirements for adults, as per the Joint FAO/WHO Expert Consultation on fats and fatty acids [6]. We also reviewed the currently available scientific evidence, primarily from population-based cohort studies and randomized control trials. These studies addressed the health impact of fats and various types of fatty acids.

Between 2007 and 2009, Korean men consumed an average of 1.25 g of ALA and 10.13 g of LA, while Korean women consumed 0.94 g of ALA and 7.01 g of LA. According to national consumption data published in 2010, the ALA intake level of Korean adults was not higher than that of other countries. It was comparable to the intake levels in the United Kingdom and Australia, and slightly higher than in France [6]. The intake levels of both EPA and DHA in Korea ranked third, following Japan and Norway, and resembled those in France [6]. Compared to Western countries, Korean adults had a lower overall fat intake, partially due to lower consumption of meat. In traditional Korean cuisine, oils are primarily used as a seasoning, rather than for pan-frying or deep-fat frying. Chinese adults, in contrast, have higher fat consumption levels than those in other Asian countries and derive more fat from vegetable sources than from animal sources [18]. Despite this, Korean adults were found to have relatively high intakes of omega-3 LC-PUFAs, such as EPA and DHA. The high consumption of fish and seafood appeared to contribute to a higher intake of PUFAs, including more highly unsaturated fatty acids relative to other countries.

Among the countries mentioned, France has the most similar natural and ecological environment to Korea, particularly in terms of food supply and production. Both countries have comparable climatic conditions and are surrounded by seas on 3 sides, providing a rich variety of fish and seafood. Consequently, the consumption of EPA and DHA in Korea resembles that in France, likely due to the shared reliance on fish and seafood, which are major sources of these fatty acids. In 2012, the annual per capita consumption of fish and seafood in Korea was 38.3 kg [19]. Meanwhile, in France, the annual consumption of fish and seafood peaked at 37 kg between 1998 and 2011, before decreasing slightly and stabilizing at around 34.5 kg from 2012 onwards [20]. A study by Welch et al. [21] found that France had the most diverse fish consumption among 10 European countries. The number of types of fish comprising 90% of the intake was highest in France, with 17 different varieties, considerably more than other European countries such as Norway, Denmark, Sweden, and the Unite Kingdom (7 types) and Germany (8 types). Similarly, the fish and seafood consumed in Korea are also quite diverse. In 2019, Koreans consumed 44 different types of fish, with 17 varieties contributing to 90% of the total fish intake [22]. Cultural factors influencing the acceptability of different fish species for consumption may also play a role in these consumption patterns. As a result, fish and seafood are the primary sources of EPA and DHA in France, contributing 72% and 65%, respectively, to the intake of these fatty acids [23]. In contrast, the intake levels of EPA and DHA in Germany, the United Kingdom, the United States, and Australia are considerably lower, as the dietary habits in these countries are culturally more focused on meat consumption rather than fish or seafood [24].

The consumption patterns of LC-PUFAs in Korea and France also exhibit notable differences. As highlighted in a prior study by Astorg et al. [23], despite substantial disparities in dietary habits, the intake of LA remained relatively consistent across various countries. For French adults, fats and oils served as the primary source of LA, accounting for one-third of the intake, similar to the pattern observed in Korea. However, in France, animal products excluding dairy also played an important role as sources of LA, a trend not seen in Korea.

Unlike LA, ALA intake seems to vary significantly across countries due to its availability in specific foods. Japan demonstrates the highest ALA intake, largely due to the prevalent use of canola and soybean oils. A prior study that compared fatty acid intake in population samples from 14 European countries found that France and other southern European countries (e.g., Greece, Italy, Portugal, and Spain) had low ALA intakes and high LA/ALA ratios [25]. This was attributed to the high consumption of LA-rich oils (e.g., sunflower oil) and the low consumption of healthy ALA sources (e.g., canola, soybean, and walnut oils) [23]. In Korea, ALA-rich oils such as soybean, perilla, and canola oils accounted for about 37% of ALA intake. The findings indicated that essential fatty acid consumption is influenced by ecological, environmental, and cultural factors, including the acceptability of different foods. Therefore, it is important to understand and critically examine the cultural aspects that influence dietary patterns, as these ultimately affect essential fatty acid consumption.

A meta-analysis of epidemiological and clinical studies indicated that the intake of EPA and DHA lowered the risk of CVD [8,9]. Despite being synthesized from ALA, EPA and DHA have distinct recommended intake guidelines, which are based on the amount needed to achieve cardioprotective effects. However, recent trends in fatty acid consumption among Korean adults reveal a marked increase in total fat and SFA intake, while PUFAs intake remains unchanged [14]. Specifically, this study highlights changes in LC-PUFAs intake, with increases in ALA and LA consumption and decreases in EPA consumption. Moreover, the shift in the food sources of fatty acid intake is noteworthy. A significant increase has occurred in the consumption of pork, beef, chicken, and eggs, while the intake of anchovy, saury, and mackerel—the primary sources of EPA and DHA—has decreased [26]. According to the KNHANES, a gradual decrease is occurring in the consumption of fish and shellfish, which are primary sources of LC-PUFAs, thereby reducing their contribution to LC-PUFAs intake [22]. The slight increase in PUFAs intake appears to be due to the presence of PUFAs in SFA food sources, as well as the increased consumption of such foods, including meat, eggs, and bread and snacks [26]. These shifts in dietary habits, along with the corresponding changes in the intake of health-protective nutrients, necessitate a thorough examination of disease patterns and major public health issues, followed by appropriate interventions.

Trends in fatty acid intake patterns are also evident in the percentage of the population adhering to global intake guidelines. These guidelines suggest a consumption of 6% or more of LC-PUFAs to prevent chronic diseases, yet most of the study population failed to reach this recommended level. On a positive note, some improvement has been made in addressing inadequate consumption. However, the intake of individual fatty acids remains unbalanced. Consequently, while the proportion of the population meeting the recommended intake of ALA and LA is on the rise, the percentage meeting the EPA+DHA recommendation is on the decline.

Furthermore, the CVD prevention benefits of EPA and DHA evident in healthy individuals are typically derived from the consumption of fish and shellfish. The effects of fish oil supplementation are primarily associated with diabetes and CVD management [27,28]. Beyond EPA and DHA, fish consumption also provides other advantageous components such as high-quality protein, which is more beneficial than the EPA and DHA obtained from basic fish oil. Consequently, both the WHO and the American Heart Association recommend consuming seafood 1-2 times/wk to meet the necessary intake of EPA and DHA for CVD prevention [29]. A strategy promoting the consumption of fish and shellfish for public health, through the adequate intake of EPA+DHA, is essential. This strategy should also be incorporated into dietary guidelines as a healthy food choice.

This study did have certain limitations. The dietary data utilized were gathered using a 1-day 24-hour recall method, which may not represent the typical intake levels of the respondents. Accordingly, the proportion of participants who did not meet the recommended intake levels may have been overestimated. The fatty acid database only became available with the 2013 KNHANES, so the LC-PUFAs intake for individuals in the 2007-2012 KNHANES was calculated based on the food content listed in the 2013 KNHANES. This could potentially differ from the actual food content present in the 2007-2012 KNHANES. Furthermore, this study was based on long-term data; thus, while interpreting the findings, it is important to consider the possibility that changes in data processing methods over the survey periods may have influenced the results. Despite these limitations, this study provides an analysis of trends in LC-PUFAs intake among a nationally representative sample of Korean adults. It offers novel insights that can be used to develop strategies for improving cardiovascular health and managing dietary risk factors for CVD morbidity and mortality.

As the risk of CVD continues to rise among Korean adults, it is crucial to assess dietary habits and monitor the intake of LC-PUFAs to develop effective dietary guidelines and health policies. The present study indicated the changes in LC-PUFAs intake trends, with an increase in the consumption of ALA and LA, but a decrease in the intake of EPA and DHA. Furthermore, the contributions of anchovy, saury, and Atka mackerel as food sources of EPA and DHA diminished over time. These findings underscore the need for strategies that promote the consumption of EPA and DHA from healthy food sources to enhance cardiovascular health in this population. Future research should explore the impact of LCPUFAs intake on health outcomes in Korean adults, utilizing data from longitudinal or clinical studies.

## Figures and Tables

**Figure f2-epih-45-e2023069:**
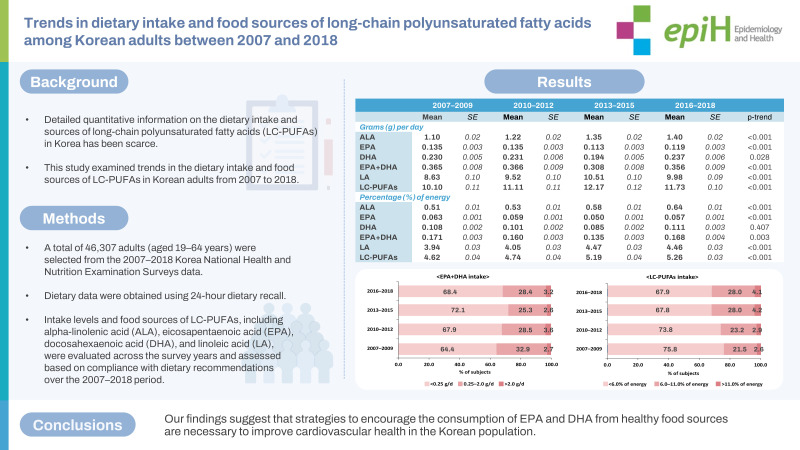


**Table 1.** Characteristics of study participants across survey years^1^

Characteristics		All (n=46,307)	2007-2009 (n=11,651)	2010-2012 (n=12,207)	2013-2015 (n=10,516)	2016-2018 (n=11,933)	p-value^2^
Gender							<0.001
	Men	52.3 (0.3)	51.9 (0.4)	51.7 (0.5)	54.1 (0.5)	51.7 (0.5)	
	Women	47.7 (0.2)	48.1 (0.4)	48.3 (0.5)	45.9 (0.4)	48.3 (0.4)	
Age (yr)							<0.001
	19-29	21.8 (0.3)	22.4 (0.4)	21.8 (0.4)	22.2 (0.4)	20.9 (0.4)	
	30-49	48.7 (0.3)	51.7 (0.5)	49.4 (0.6)	49.1 (0.5)	45.4 (0.5)	
	50-64	29.6 (0.3)	25.9 (0.3)	28.8 (0.3)	28.8 (0.4)	33.7 (0.4)	
Area of residence							0.178
	Urban	84.1 (0.7)	82.9 (1.0)	82.3 (1.1)	84.1 (1.0)	86.5 (1.0)	
	Rural	15.9 (0.6)	17.1 (0.7)	17.7 (0.8)	15.9 (0.8)	13.5 (0.7)	
Household income							<0.001
	Lowest	10.0 (0.2)	11.0 (0.2)	10.3 (0.3)	9.3 (0.2)	9.8 (0.3)	
	Medium-low	25.0 (0.3)	25.3 (0.4)	27.4 (0.4)	24.4 (0.4)	23.1 (0.4)	
	Medium-high	31.5 (0.3)	30.9 (0.4)	32.0 (0.5)	31.7 (0.5)	31.4 (0.5)	
	Highest	33.4 (0.4)	32.8 (0.5)	30.3 (0.5)	34.6 (0.6)	35.7 (0.6)	
Energy and macronutrient intake levels							
	Total energy (kcal)	2,068.6±5.5	1,966.2±10.4	2,100.7±11.1	2,154.0±10.5	2,037.1±11.0	<0.001
	Carbohydrate (% of energy)	64.3±0.1	66.4±0.2	65.2±0.2	63.8±0.1	62.2±0.1	<0.001
	Fat (% of energy)	20.6±0.1	18.6±0.1	19.7±0.1	21.3±0.1	22.3±0.1	<0.001
	Protein (% of energy)	15.1±0.0	15.0±0.1	15.1±0.1	14.9±0.1	15.5±0.1	<0.001

Values are presented as % (standard error) for categorical variables and mean±standard error for continuous variables.

1 The statistical analysis accounted for the complex sampling design effect and included an appropriate sample weight.

2 From the chi-square test for categorical variables and regression analysis for continuous variables.

**Table 2.** Trends in the intake (g) of LC-PUFAs from 2007 to 2018^1^

Variables	All				p-trend^2^	Men				p-trend^2^	Women				p-trend^2^
	2007-2009	2010-2012	2013-2015	2016-2018		2007-2009	2010-2012	2013-2015	2016-2018		2007-2009	2010-2012	2013-2015	2016-2018	
ALA	1.10±0.02	1.22±0.02	1.35±0.02	1.40±0.02	<0.001	1.25±0.02	1.37±0.02	1.45±0.02	1.53±0.02	<0.001	0.94±0.02	1.07±0.02	1.24±0.02	1.26±0.02	<0.001
EPA	0.135±0.003	0.135±0.003	0.113±0.003	0.119±0.003	<0.001	0.165±0.006	0.166±0.005	0.134±0.004	0.138±0.004	<0.001	0.103±0.003	0.102±0.003	0.089±0.003	0.098±0.003	<0.001
DHA	0.230±0.005	0.231±0.006	0.194±0.005	0.237±0.006	0.028	0.280±0.008	0.282±0.009	0.231±0.008	0.280±0.010	0.011	0.175±0.005	0.176±0.005	0.151±0.005	0.191±0.006	0.809
EPA+DHA	0.365±0.008	0.366±0.009	0.308±0.008	0.356±0.009	<0.001	0.445±0.014	0.448±0.014	0.365±0.012	0.418±0.014	<0.001	0.278±0.007	0.278±0.008	0.240±0.008	0.289±0.009	0.163
LA	8.63±0.10	9.52±0.10	10.51±0.10	9.98±0.09	<0.001	10.13±0.15	11.13±0.15	11.67±0.15	11.41±0.13	<0.001	7.01±0.09	7.80±0.10	9.14±0.12	8.45±0.09	<0.001
LC-PUFAs	10.10±0.11	11.11±0.11	12.17±0.12	11.73±0.10	<0.001	11.82±0.17	12.95±0.17	13.49±0.17	13.35±0.15	<0.001	8.23±0.10	9.15±0.12	10.62±0.14	10.00±0.10	<0.001

Values are presented as mean±standard error. LC-PUFAs, long-chain polyunsaturated fatty acids; ALA, alpha-linolenic acid; EPA, eicosapentaenoic acid; DHA, docosahexaenoic acid; LA, linoleic acid.

1 The statistical analysis accounted for the complex sampling design effect and included an appropriate sample weight.

2 From a multiple linear regression model after adjusting for gender, age, area of residence, household income, and total energy intake, where applicable.

**Table 3.** Trends in the percentage (%) of energy from LC-PUFAs from 2007 to 2018^1^

Variables	All				p-trend^2^	Men				p-trend^2^	Women				p-trend^2^
	2007-2009	2010-2012	2013-2015	2016-2018		2007-2009	2010-2012	2013-2015	2016-2018		2007-2009	2010-2012	2013-2015	2016-2018	
ALA	0.51±0.01	0.53±0.01	0.58±0.01	0.64±0.01	<0.001	0.51±0.01	0.53±0.01	0.56±0.01	0.61±0.01	<0.001	0.51±0.01	0.54±0.01	0.61±0.01	0.66±0.01	<0.001
EPA	0.063±0.001	0.059±0.001	0.050±0.001	0.057±0.001	<0.001	0.069±0.002	0.065±0.002	0.054±0.002	0.059±0.002	<0.001	0.057±0.001	0.053±0.001	0.045±0.001	0.054±0.002	0.007
DHA	0.108±0.002	0.101±0.002	0.085±0.002	0.111±0.003	0.407	0.118±0.003	0.110±0.003	0.092±0.003	0.117±0.004	0.075	0.007±0.002	0.091±0.003	0.076±0.002	0.105±0.003	0.425
EPA+DHA	0.171±0.003	0.160±0.003	0.135±0.003	0.168±0.004	0.003	0.187±0.005	0.175±0.005	0.146±0.004	0.176±0.006	0.001	0.154±0.004	0.144±0.004	0.121±0.003	0.159±0.005	0.720
LA	3.94±0.03	4.05±0.03	4.47±0.03	4.46±0.03	<0.001	4.10±0.04	4.20±0.04	4.48±0.04	4.51±0.04	<0.001	3.77±0.04	3.89±0.04	4.45±0.04	4.40±0.03	<0.001
LC-PUFAs	4.62±0.04	4.74±0.04	5.19±0.04	5.26±0.03	<0.001	4.80±0.05	4.90±0.05	5.19±0.05	5.29±0.04	<0.001	4.44±0.04	4.57±0.05	5.18±0.05	5.23±0.04	<0.001

Values are presented as mean±standard error. LC-PUFAs, long-chain polyunsaturated fatty acids; ALA, alpha-linolenic acid; EPA, eicosapentaenoic acid; DHA, docosahexaenoic acid; LA, linoleic acid.

1 The statistical analysis accounted for the complex sampling design effect and included an appropriate sample weight.

2 From a multiple linear regression model after adjusting for gender, age, area of residence, and household income, where applicable.

**Table 4.** Changes in food sources of long-chain polyunsaturated fatty acids from 2007-2009 to 2016-2018

Rank		All				Men				Women			
		2007-2009		2016-2018		2007-2009		2016-2018		2007-2009		2016-2018	
		Food item	%^1^	Food item	%^1^	Food item	%^1^	Food item	%^1^	Food item	%^1^	Food item	%^1^
Alpha-linolenic acid													
	1.	Soybean oil	25.6	Soybean oil	17.4	Soybean oil	28.6	Soybean oil	20.4	Soybean oil	22.9	Perilla oil	16.5
	2.	Perilla oil	8.8	Perilla oil	15.0	Perilla oil	6.9	Perilla oil	13.3	Perilla oil	10.6	Soybean oil	14.8
	3.	Soybean	7.3	Mayonnaise	11.7	Tofu	6.9	Mayonnaise	11.3	Soybean	7.7	Mayonnaise	12.0
	4.	Mayonnaise	6.7	Perilla seed	6.6	Soybean	6.8	Perilla seed	5.7	Mayonnaise	7.1	Perilla seed	7.3
	5.	Tofu	6.5	Tofu	4.9	Mayonnaise	6.2	Tofu	5.3	Tofu	6.2	Canola oil	4.7
	6.	Perilla seed	5.8	Canola oil	4.3	Perilla seed	5.7	Pork	4.5	Perilla seed	5.9	Tofu	4.6
	7.	Soybean paste	4.2	Pork	3.6	Pork	4.3	Canola oil	3.9	Soybean paste	4.4	Walnut	3.1
	8.	Pork	3.6	Soybean	2.8	Soybean paste	4.0	Soybean	2.8	Pork	2.9	Soybean	2.8
	9.	Canola oil	2.1	Walnut	2.6	Canola oil	1.8	Egg	2.3	Canola oil	2.5	Pork	2.7
	10.	Perilla leaf	1.3	Egg	2.3	Bean sprouts	1.4	Walnut	2.0	Perilla leaf	1.5	Egg	2.3
Eicosapentaenoic acid													
	1.	Mackerel	19.5	Mackerel	23.0	Mackerel	20.1	Mackerel	25.2	Mackerel	18.7	Mackerel	20.7
	2.	Anchovy	17.5	Anchovy	7.6	Anchovy	17.2	Anchovy	7.2	Anchovy	17.9	Anchovy	8.0
	3.	Saury	8.0	Laver	6.5	Saury	7.7	Eel	6.8	Saury	8.2	Laver	7.6
	4.	Laver	5.1	Eel	6.4	Atka mackerel	5.6	Laver	5.6	Laver	6.1	Eel	6.0
	5.	Squid	4.3	Squid	5.1	Laver	4.2	Squid	5.1	Squid	4.7	Squid	5.0
	6.	Atka mackerel	4.2	Croaker	4.2	Squid	4.0	Croaker	4.7	Hairtail	4.3	Pollack	4.7
	7.	Hairtail	4.1	Saury	4.1	Hairtail	3.9	Saury	4.6	Eel	3.6	Oyster	3.6
	8.	Eel	3.6	Pollack	3.8	Eel	3.5	Oyster	3.1	Salted seafood	3.6	Croaker	3.6
	9.	Salted seafood	3.4	Oyster	3.4	Salted seafood	3.3	Pollack	3.0	Pollack	3.2	Saury	3.6
	10.	Oyster	3.2	Hairtail	2.9	Oyster	3.2	Hairtail	2.7	Oyster	3.1	Hairtail	3.2
Docosahexaenoic acid													
	1.	Mackerel	23.0	Mackerel	25.3	Mackerel	24.0	Mackerel	26.7	Mackerel	21.9	Mackerel	23.8
	2.	Anchovy	14.2	Squid	9.4	Anchovy	13.8	Squid	9.4	Anchovy	14.6	Squid	9.4
	3.	Saury	9.9	Eel	7.5	Saury	9.6	Eel	7.5	Saury	10.3	Egg	7.8
	4.	Squid	7.2	Egg	7.0	Squid	6.6	Egg	6.2	Squid	7.9	Eel	7.4
	5.	Hairtail	6.2	Anchovy	5.3	Hairtail	5.9	Anchovy	4.9	Hairtail	6.6	Anchovy	5.6
	6.	Eel	5.1	Saury	4.4	Eel	4.9	Saury	4.9	Eel	5.2	Hairtail	4.3
	7.	Croaker	3.8	Croaker	4.3	Croaker	3.4	Croaker	4.8	Croaker	4.1	Saury	3.8
	8.	Salted seafood	3.4	Hairtail	3.8	Salted seafood	3.3	Flatfish	3.8	Salted seafood	3.5	Croaker	3.7
	9.	Pollack	2.9	Flatfish	3.1	Pollack	2.9	Hairtail	3.5	Pollack	3.0	Pollack	3.6
	10.	Tuna	2.4	Pollack	2.9	Atka mackerel	2.5	Tuna	2.3	Tuna	2.5	Flatfish	2.4
Linoleic acid													
	1.	Soybean oil	22.5	Soybean oil	18.4	Soybean oil	24.1	Soybean oil	20.4	Soybean oil	20.9	Soybean oil	16.4
	2.	Sesame oil	8.7	Mayonnaise	11.8	Pork	9.9	Mayonnaise	10.8	Sesame oil	8.8	Mayonnaise	12.8
	3.	Pork	8.6	Pork	7.7	Sesame oil	8.5	Pork	9.3	Tofu	7.4	Sesame oil	6.8
	4.	Tofu	7.5	Sesame oil	6.7	Tofu	7.6	Sesame oil	6.6	Pork	7.2	Pork	6.2
	5.	Soybean	6.5	Egg	5.0	Soybean	5.8	Tofu	4.9	Mayonnaise	7.1	Egg	5.3
	6.	Mayonnaise	6.4	Tofu	4.9	Mayonnaise	5.8	Egg	4.8	Soybean	7.1	Tofu	4.9
	7.	Ramyeon	3.5	Bread	2.9	Ramyeon	4.0	Ramyeon	3.1	Ramyeon	3.0	Bread	3.2
	8.	White rice	2.7	Ramyeon	2.6	White rice	2.7	Bread	2.6	White rice	2.8	Soybean	2.8
	9.	Egg	2.5	Soybean	2.6	Egg	2.5	Soybean	2.4	Egg	2.5	Snacks, biscuits & cookies	2.1
	10.	Sesame seed	1.9	Snacks, biscuits & cookies	2.0	Sesame seed	1.9	Snacks, biscuits & cookies	1.9	Bread	2.3	Ramyeon	2.1

*Ramyeon*, instant noodle.

1 Contribution %.
